# Supplementary material for: Amyloid and Tau Protein Concentrations in Children with Meningitis and Encephalitis
Source: Viruses. 2022 Mar 30;14(4):725. doi: 10.3390/v14040725 (PMC9027807; doi:10.3390/v14040725)
Supplement: Supplementary file 1 [file viruses-14-00725-s001.zip › viruses-1616385-supplementary.pdf]

Table S1. Ratios of cerebrospinal fluid biomarkers of Alzheimer's disease in study groups

	Enteroviruses (n=12)	Varicella zoster virus (n=5)	Tick-borne encephalitis virus (n=9)	Bacterial – purulent (n=8)	Bacterial – aseptic (n=11)	Controls (n=8)
total tau/amyloid β_{1-42}	0.283 (0.208-0.494)	0.741 (0.325-0.821)	0.438 (0.416-0.447)	1.03 (0.384-1.383)[1,5,6]	0.275 (0.219-0.65)[4]	0.292 (0.223-0.456)[4]
total tau/ amyloid β_{1-40}	0.025 (0.017-0.039)[4]	0.042 (0.031-0.064)[4]	0.038 (0.022-0.042)[4]	0.068 (0.026-0.217)[1,2,3,5,6]	0.02 (0.016-0.029)[4]	0.023 (0.018-0.028)[4]
phosphorylated tau/ amyloid β_{1-42}	0.051 (0.038-0.091)	0.049 (0.049-0.054)	0.062 (0.047-0.087)	0.108 (0.082-0.169)	0.075 (0.047-0.116)	0.046 (0.041-0.086)
phosphorylated tau/ amyloid β_{1-40}	0.005 (0.003-0.008)[4]	0.004 (0.003-0.005)[4]	0.005 (0.004-0.005)[4]	0.01 (0.006-0.015)[1,2,3,5,6]	0.005 (0.004-0.005)[4]	0.004 (0.003-0.005)[4]

Data are presented as medians with interquartile range. 1 indicates $p<0.05$ when compared with enteroviruses; 2 indicates $p<0.05$ when compared with varicella zoster virus; 3 indicates $p<0.05$ when compared with tick-borne encephalitis virus; 4 indicates $p<0.05$ when compared with purulent meningitis/encephalitis; 5 indicates $p<0.05$ when compared with bacterial – aseptic (Lyme neuroborreliosis); 6 indicates $p<0.05$ when compared with the control group.

Table S2. Cerebrospinal fluid concentrations of biomarkers of Alzheimer's disease divided by type of the CNS infection

	Aseptic (n=37)	Purulent (n=8)	Controls (n=8)
**amyloid β_{1-40} (pg/mL)**	8998.2 (5477.6-12550.8)	7776.8 (5749.2-9145.9)[3]	13208.8 (8868.5-15717.4)[2]
**amyloid β_{1-42} (pg/mL)**	549.5 (381.4-955.2)	558.7 (470.1-756.6)	833.2 (546.6-1370.1)
**amyloid β42/40**	0.068 (0.054-0.096)	0.075 (0.054-0.095)	0.074 (0.053-0.091)
**total tau (pg/mL)**	236.7 (133.6-313)[2]	562 (180.1-1331.3)[1,3]	250.7 (217.7-340.3)[2]
**phosphorylated tau (pg/mL)**	41 (25.9-52.2)[2]	66.4 (41.3-109.2)[1,2]	47.5 (35.2-58.5)[2]
**total tau/amyloid β_{1-42}**	0.354 (0.227-0.65)[2]	1.03 (0.384-1.383)[1,3]	0.292 (0.223-0.456)[2]
**total tau/ amyloid β_{1-40}**	0.026 (0.019-0.041)[2]	0.068 (0.026-0.217)[1,3]	0.023 (0.018-0.028)[2]
**phosphorylated tau/ amyloid β_{1-42}**	0.053 (0.046-0.095)[2]	0.108 (0.082-0.169)[1,3]	0.046 (0.041-0.086)[2]
**phosphorylated tau/ amyloid β_{1-40}**	0.005 (0.004-0.006)[2]	0.01 (0.006-0.015)[1,3]	0.004 (0.003-0.005)[2]

The aseptic group comprises children with viral infections of the CNS and children with Lyme neuroborreliosis (n=37). The purulent group comprises children with purulent bacterial infections of the CNS (n=8). Data are presented as medians with interquartile range. 1 indicates $p<0.05$ when compared with aseptic meningitis/encephalitis; 2 indicates $p<0.05$ when compared with purulent meningitis/encephalitis; 3 indicates $p<0.05$ when compared with the control group

Table S3. Cerebrospinal fluid concentrations of biomarkers of Alzheimer's disease divided by the severity of the CNS infection

	**Meningitis (n=16)**	**Encephalitis (n=10)**	**Controls (n=8)**
**amyloid $\beta_{1\text{-}40}$ (pg/mL)**	9721.8 (8113.1-13087.1)	8217.9 (5477.6-15482.1)	13208.8 (8868.5-15717.4)
**amyloid $\beta_{1\text{-}42}$ (pg/mL)**	712.4 (432.3-1030.8)	817.2 (341.8-1227.7)	833.2 (546.6-1370.1)
**amyloid β42/40**	0.058 (0.051-0.089)[2]	0.097 (0.086-0.101)[1]	0.074 (0.053-0.091)
**total tau (pg/mL)**	243.5 (174.1-314.4)	257.2 (123-527.5)	250.7 (217.7-340.3)
**phosphorylated tau (pg/mL)**	41 (26-50.4)	41.5 (26.1-87.9)	47.5 (35.2-58.5)
**total tau/amyloid $\beta_{1\text{-}42}$**	0.37 (0.208-0.92)	0.423 (0.354-0.447)	0.292 (0.223-0.456)
**total tau/ amyloid $\beta_{1\text{-}40}$**	0.025 (0.017-0.042)	0.037 (0.029-0.044)	0.023 (0.018-0.028)
**phosphorylated tau/ amyloid $\beta_{1\text{-}42}$**	0.051 (0.038-0.107)	0.058 (0.049-0.076)	0.046 (0.041-0.086)
**phosphorylated tau/ amyloid $\beta_{1\text{-}40}$**	0.004 (0.003-0.006)	0.005 (0.004-0.006)	0.004 (0.003-0.005)

In the comparison children with viral infections of the CNS were included only. The meningitis group (n=16) comprises children with enteroviral meningitis (n=10), varicella zoster virus meningitis (n=3), tick-borne encephalitis (n=3). The encephalitis group (n=10) comprises children with enteroviral encephalitis (n=2), varicella zoster viral encephalitis (n=2), and tick-borne encephalitis (n=6). Data are presented as medians with interquartile range. 1 indicates $p<0.05$ when compared with meningitis; 2 indicates $p<0.05$ when compared with encephalitis
